# Editorial

**Published:** 1860-08

**Authors:** 


### Editorial.

### BICUSPID PIVOT TEETH.

It is a general rule that the six anterior teeth only are susceptible of pivot teeth. The roots of bicuspid teeth, especially when much compressed, or terminating in two fangs, have not been used for the insertion of pivot teeth. But by care and a very simple arrangement, they are susceptible of as perfect operation as the roots of any of the anterior teeth.

For illustration we will describe a case: Mrs. L. presented the left superior bicuspid, with the crown principally decayed away; it had been filled, on both sides, large cavities, and the fang partially filled, as the decay had extended somewhat into it. The root was considerably compressed, and probably terminates in two fangs. The remaining portion of the crown was removed, and the root dressed as usual for a pivot tooth, then removed the gold that had been introduced into the root, and cleansed out the decayed cavity, which was not very large. Then with a drill that measured 20 stubs gauge, passed into the canal at its buccal termination, and also at its labial termination, thus making two small holes in the root instead of one large one. A hole through the center of the fang as large as an ordinary pivot would have divided it. These two holes are about a line asunder, and about three lines deep; they should be made perfectly parallel, which may be done by inserting a wire into the first one, and in drilling the second keep the drill parallel with the wire.

Two wires of the same size as the drill were placed in the cavities, and protruded some two or three lines. Having previously prepared the decayed cavity properly, proceeded to fill, with the wires in place, using adhesive gold; fill the cavity perfectly, then remove the wires, and dress the filling with the articulating surface of the fang. Then dress the wires so they will pass readily into the holes, which being done, take an impression of the part in plaster; the wires will come away with the impression, then get a model from this; there will then be, upon the removal of the wires, the exact position of the holes of the root, in the model, which will be an exact guide in attaching the pivots.

From this model get a metallic model and counter model ; then swage a piece of plate to fit, and large enough to cover the articulating surface of the root. Then perforate it for the two pivots ; this plate should be laid upon the model, and the pivots passed through it down into the holes in the model already described, and soldered to the plate while in this position. It may then be dressed up for the reception of the crown. This should be an ordinary plate bicuspid tooth, selected with reference to size, shade, and articulation with the opposing teeth. The tooth is then ground to fit the plate perfectly, jutting slightly over it at the labial edge. The plate is then placed on the root, and the tooth adjusted and attached with wax, then remove all together, invest, the tooth back and solder as usual, then finish, and insert. It will pass readily to its place, and maintain its position firmly. It can not rotate or slip out of place, and yet can be readily removed, can be used as well as a natural tooth in mastication ; the pivots will not mar the root ; it can be kept perfectly clean. It sets much more firm than a single pivot crown can. If desirable, it can be easily tightened by a slight spring of the pivots. T.

☞ We still use the mallet more than ever, and are more and more pleased with it ; many things can be done with it that can not be done by hand pressure. No one who will use it properly can fail to be pleased with it. We may have something to say on the subject in the next number. T.

### A SINGULAR SPECIMEN.

We have received of Dr. D. L. Pratt, for the College Museum, a rather singular specimen, which we will describe in his own language. "It is a piece of the shin bone of an ox, which I was sawing for mechanical purposes, when I discovered a grain of shot imbedded in the bone, and completely surrounded by it." This has evidently been shot into the leg of the animal, probably when it was young, and bone material thrown out over it so as to completely imbed the lead in the bone. The bone is about alike perfect on all sides of the shot. Lead remains imbedded in living tissue with perfect impunity ; we know of no other metal that will be thus tolerated. T.

PERSONALITIES.

Personalities, precipices, whirlpools and whisky are things to be avoided. It may be dangerous, even, to write about them. However, we will venture a few remarks on the first mentioned.

We are decidedly of the opinion that both writers and speakers should in general "avoid personalities," but we are as well convinced that many who inculcate this sage doctrine have no true idea of its meaning. Many regard it as highly proper to refer to the African gentleman among the fuel, but grossly personal to allude to "the nigger in the wood pile."

We are of the opinion that many complain of personality without any cause. A man writes an article and *publishes* it, and thus renders it public property, as to its language and sentiment ; yet we often find he regards the article as a part and parcel of himself, and practically maintains that whosoever touches it, touches "the apple of his eye." Now we maintain that when we publish an article, any one has a right to object to, or criticise, every word, every letter, every comma, semicolon or period, and that we have no reason to complain if he does, and have still less right to charge him with personality for so doing. If the criticisms are unjust, we are not worsted by them, nor is our article ; if they are just, we are benefited, and our article is merely rendered less capable of doing harm. An article that will not bear such examination should not have been written ; and being written, it should be consigned to the oblivion it merits.

The great want of the world is genuine, manly frankness. In the common intercourse of life, in speaking, and especially in writing, it is refreshing to find it manifested. Its introduction should be hailed as a good omen. It should be encouraged and cultivated. It is more manly, and no less friendly, than the fashionable circumlocution of those who are so sensitive on the subject.

Two noted men once met at Antioch. The one thought the other was in fault, and "withstood him to the face," saying, "If thou, being a Jew, livest after the manner of the Gentiles, and not as do the Jews, why compellest thou the Gentiles to live as do the Jews." Now, if he had held the views of some now living, he would not have "withstood him to the *face;*"—that was personal. He would have turned from him and said to the audience, "No man can have a better opinion of the gentleman from Jerusalem than myself. He is a great and good man; but I am almost afraid that, for want of thought, (not that I blame him in the least for it,) he has been led into a slight inconsistency, in that he advises those of other nations to observe the rites and customs of the descendants of Abraham, while he, though an Israelite, does not observe them himself."

When Paul wanted to talk *to* Peter, he did it; and afterward, when he wanted to talk *about* him, he did that. W.

### SELF SATISFIED.

"I am satisfied, after using them in my practice, successfully, for more than twenty years, that I have attained the desideratum sought."—*Sanford—Cathartic Pills.*

"In short, through the assistance of some superior power, she is endowed with the healing art."—*Miss Tennessee Claflin, the Planet Reader.*

"The madam has valuable remedies of her own discovery for healing the sick and afflicted of all manner of diseases."—*Madam Virginia, the great Russian Prophetess.*

"Again I present my almanac to the public,—I would call the particular attention of every reader to the strengthening cordial—the greatest remedy in the world. The principal ingredient is known only to myself."—*McLean's Almanac.*

"Our journal has already attained such a position, that we feel justified in claiming it to be the representative of the Science and Art of Dentistry."—*Dental Cosmos.* W.

